# Long-term clinical and radiological outcomes of a stemless reverse shoulder implant that is fallen out of favor - stemless nano-reverse shoulder arthroplasty

**DOI:** 10.1186/s12891-025-09386-1

**Published:** 2026-01-23

**Authors:** Johannes E. Plath, Nicolas Saiczek, Edgar Mayr, Christian Schoch, Johann Wasmaier, Wolfgang Vogt

**Affiliations:** 1 Orthopädisches Fachzentrum (OFZ), Johann-Baur Str. 5, Weilheim, 82362 Deutschland; 2 Klinik für Unfallchirurgie, Orthopädie, Plastische und Handchirurgie, Universitätsklinikum Augsburg, Augsburg, Deutschland; 3 St. Vinzenz Klinik Pfronten, Pfronten, Deutschland; 4 Vogt Ortho Consulting and Development, Garmisch-Partenkirchen, Deutschland

**Keywords:** Stemless reverse shoulder arthroplasty, Comprehensive nano reverse shoulder arthroplasty system, Long-term follow-up, Onlay reverse total shoulder system

## Abstract

**Background:**

The purpose of this study was to assess the long-term results of the stemless onlay Comprehensive Nano reverse total shoulder arthroplasty (rTSA) system.

**Methods:**

We evaluated 35 shoulders at an average follow-up of 106 ± 14.6 months (range, 80–135) (follow-up rate 66%). Patients were evaluated via the Constant–Murley scale, the DASH score (Disabilities of the Arm, Shoulder and Hand) and the subjective shoulder value (SSV). Furthermore, a visual analog scale (VAS) for pain intensity was used. The passive glenohumeral range of motion and active total range of motion were recorded.

Radiographic assessment was performed on true antero-posterior and axillary views.

**Results:**

The mean age at surgery was 72.8 ± 6.7 years (range, 47–82). Four patients were revised to a stemmed implant during the follow-up period and were excluded from further assessment. In the remaining group, the Constant score was 82.9 ± 13.1 (range, 40–97), the DASH score was 10.6 ± 17.3 (range, 0–77.5), the SSV was 85.0 ± 18.1 (range, 10–100), and the VAS score for pain was 0.9 ± 1.7/10 (range, 0–7).

The mean active flexion and abduction values were 159.8 ± 13.8 and 155.9 ± 20.1, whereas the active external and internal rotation values averaged 34.8 ± 15.3 and 88.9 ± 7.9, respectively.

Grade I radiolucency lines (RLLs) were found in 4 patients (14.3%). RLLs > 1 mm were not observed. Two patients experienced early varus displacement of the humeral tray with full reintegration without revision. Revisions to a stemmed implant were performed for atraumatic peg breakage of the humeral tray in 2 patients, early septic loosening in one patient and periprosthetic fracture in one patient.

Grade 1 notching was found in 17.9%, and acromion stress fracture was found in 3.6%. Three patients experienced postoperative neurological deficits, with complete recovery in 2 patients.

**Conclusions:**

Compared with published data on stemmed and stemless rTSA, the comprehensive Nano rTSA system in the present study has comparable or even superior clinical outcomes at long-term follow-up. The rates of implant-associated complications and revision, however, are high compared with those reported in the literature.

**Trial registration:**

Retrospectively registered on 6th August 2025 German Clinical Trial Register, clinical trial number DRKS00037624, https//www.drks.de/DRKS00037624.

## Introduction

Reverse total shoulder arthroplasty (rTSA) is a reliable and well-established surgical option for many shoulder conditions ranging from cuff tear arthropathy to irreparable rotator cuff tears without arthritic changes, chronic glenohumeral instability, primary osteoarthritis with eccentric wear or a “cuff at risk” and proximal humerus fracture. Consequently, the number of rTSA implantations has rapidly increased in recent decades [1]. As the number of rTSA implantations increases, so does the understanding of implant biomechanics, fixation techniques and potential associated complications, which in turn has led to implant innovations and modifications to the initial Grammont concept in recent decades [2–6]. 

One of the most recent innovations on the humeral side has been the introduction of stemless rTSA implants. Since the introduction of the first stemless implant to the European market in 2005 (Total Evolutive Shoulder System (TESS), Biomet France), an increasing number of medical device manufacturers have offered stemless rTSA implants [7]. 

The primary idea of stemless shoulder arthroplasty is the preservation of diaphyseal bone stock by metaphyseal implant fixation, which yields potential advantages over stemmed fixation. Despite the use of a potentially simplified technique and lower blood loss when the diaphysis is left alone, the greatest advantage of stemless TSA is the improved bony condition for potentially necessary revision in the future. In particular, in view of the abovementioned increasing number of implantations, the incidence of revision shoulder arthroplasty is expected to increase as well. A further advantage is the increased loading of the metaphyseal bone, which may reduce stress shielding, which is a concern in traditional humeral components and is found in more than 80% of cases [8]. Finally, stemless implants allow the surgeon to implant the humeral component in the desired position, version and inclination regardless of the shape and orientation of the diaphysis, which may especially be advantageous in fracture sequelae and deformities.

While the use of a stemless design is well established in anatomic total shoulder arthroplasty (aTSA) and supported by a sufficient number of clinical long-term follow-up clinical studies, there is a paucity of long-term data on the use of stemless rTSA [9–14]. 

For stemmed rTSA humeral implants, stemless implant designs can be categorized as “inlay” or “onlay” implants. Most stemless rTSA implants on the market follow an “inlay” design where the implant is completely embedded into the metaphyseal bone below the humeral resection level. Biomechanically, “inlay” implants have an advantage because the forces are transferred directly to the bone, whereas “onlay” systems have a longer lever arm with a potential risk of seesaw motions [7]. 

In 2012, the Comprehensive Nano system (Zimmer Biomet, Warsaw, USA) was launched on the market. This modular system was based on the Nano anchor, which was fixed to the metaphyseal bone by six thick hydroxyapatite-coated fins and could be used in a reverse “onlay” as well as an anatomic configuration. The reverse version, however, was withdrawn from the market after five years of use due to insufficient fixation and early loosening in some cases. To date, only a single study on the Comprehensive Nano rTSA system has reported the clinical and radiological outcomes of 15 patients at an average follow-up of 27 months [15]. In this series, 4 out of 15 implants were revised to stemmed implants during follow-up, supporting the decision of the company.

We have used the Comprehensive Nano rTSA system in selected cases since its introduction to the European market in 2012 until its withdrawal in 2017.

The aim of this study was to assess the clinical and radiological results of the stemless Comprehensive Nano rTSA “Onlay” system at long-term follow-up. Our hypothesis was that this design would provide good long-term clinical and radiological results.

## Methods

Between 2012 and 2017, rTSA using the stemless Comprehensive Nano system was performed in 53 shoulders (52 patients) at the Orthopedic Specialist Center in Weilheim, Germany.

The indications for performing rTSA were irreparable rotator cuff tears, cuff tear arthropathy and primary as well as secondary osteoarthritis. The contraindications for stemless rTSA were poor bone quality during intraoperative assessment, large metaphyseal cysts on preoperative imaging, a history of osteoporosis, long-term corticosteroid intake and chronic renal insufficiency. Advanced age was not considered a contraindication for stemless rTSA. Preoperatively, patients underwent shoulder CT scans with 3D reconstruction of the scapula for preoperative planning (Biomet Signature One Surgical Planning).

All patients were contacted by telephone and enrolled in the study. Only patients with a follow-up of greater than 80 months were included in the study.

Ethical approval for this study protocol was granted by our local ethics committee board (IRB approval No. 23/0954). The consent of each patient was obtained.

### Surgical technique and rehabilitation

All surgeries were performed by the senior author. Patients were operated on in the beach chair position under single-shot antibiotic prophylaxis via a standard deltoideo-pectoral approach. The subscapularis tendon was tenotomized, and the humeral head was exposed. The humeral head cut was performed freehand. Version and inclination were adapted to the specific patient anatomy. Typically, an anatomic resection angle of approximately 135° and a retrotorsion angle between 10° and 40° were aimed for.

After the humeral head cut the bone quality was assessed using the “thumb test”, and in combination with the preoperative CT scan, the indication for stemless rTSA was considered. The humeral cut was protected with a plate.

The base plate and glenosphere were implanted in a standardized fashion. Typically, the baseplate was fixed with a central 6.5 mm screw and two fixed-angle 4.75 mm peripheral screws for fixation with a slight inferior tilt at neutral version.

Using a humeral sizer, the correct size of the humeral Nano component was selected, aiming for a remaining cancellous ring of approximately 5 mm. After the insertion of a Steinman pin, the resection surface was reamed and broached.

Before implantation of the definite humeral implant, transosseous Ethibond sutures (Ethicon/J&J, Bridgewater, NJ, USA) were passed through the bicipital groove for subscapularis tendon refixation. The definitive stemless Nano implant was impacted. A trial reverse liner was used to assess soft-tissue tension and potential notching at the scapular neck, acromion or coracoid. The definitive humeral tray and bearing were implanted, the shoulder was reduced, and the subscapularis tendon was repaired.

The shoulder was immobilized on a sling for 2–5 days after surgery and at night for 2 weeks.

Physiotherapy started one day after surgery, with passive and assistive mobilization for 4 weeks and active mobilization beginning at week 5 after surgery. Shoulder strengthening was not allowed before week 10.

### Clinical outcome measures

Patients were evaluated via the Constant–Murley scale and DASH score (Disabilities of the Arm, Shoulder and Hand) [16, 17]. Strength measurements were performed during abduction via a digital hand-held dynamometer (Lafayette Instrument Company, Lafayette, Indiana, USA). All patients were further asked to rate their global shoulder function from 0% to 100% in 5% increments compared to a normal shoulder (subjective shoulder value - SSV) and on a visual analog scale (VAS) for pain (0 representing no pain and 10 representing maximal pain) [18, 19]. 

Furthermore, the passive glenohumeral range of motion and active total range of motion were recorded. Score assessment and physical examination were carried out by a board-certified orthopedic surgeon specializing in shoulder surgery.

### Diagnostic imaging

**Fig. 1:**
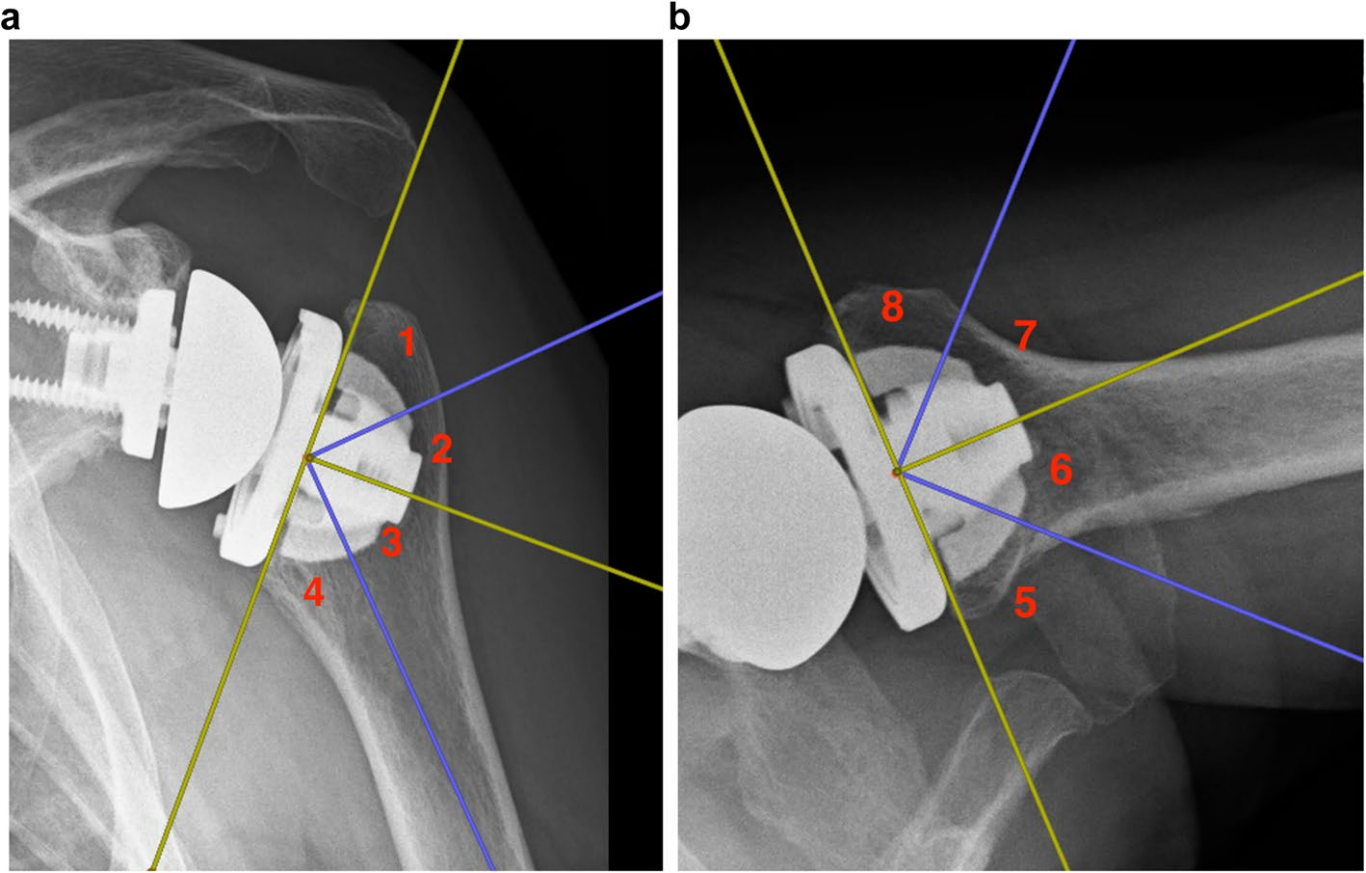
For radiological assessment, the humerus was divided into 8 distinct zones on antero-posterior (zones 1--4) and axillary (zones 5--8) X-rays according to Moroder et al. [20]

For radiographic assessment, a true antero-posterior and an axillary view were taken at follow-up and judged by three board-certified orthopedic surgeons in consensus. Radiolucency was rated according to the classification of Moroder et al. [20] In their publication, the authors defined 8 distinct zones, zones 1–4 on true antero-posterior view and zones 5–9 on the axillary view. Each zone corresponds to an angle of 45° from the bone–implant interface. (Fig. 1a/b) Radiolucency was judged as 0–no sclerosis, 1–sclerosis less than 1 mm, 2–sclerosis more than 1 mm in space and 3–implant loosening.

In addition to radiolucency, all images were assessed for scapular notching, bone resorption and acromion and spine insufficiency fractures. Postoperative X-rays were available for comparison.

### Statistics

Statistical analyses were performed via SPSS software version 30 for Mac (SPSS Inc., Chicago, Illinois, USA). A descriptive data analysis was conducted for the current study.

## Results

The clinical data of 35 shoulders in 34 patients (16 females/18 males) were obtained from 53 shoulders that were treated with a Comprehensive Nano stemless rTSA system and were eligible for this long-term follow-up study (follow-up rate 66%). However, 4 shoulders (2 females/2 males) were revised to a stemmed implant during the follow-up period and excluded from further assessment, and in 3 patients, only a telephone interview could be performed. Ten patients died, 7 patients could not be reached, and 3 patients were immobile and/or suffered from advanced dementia. The mean age of the included patients at the time of surgery was 72.8 ± 6.7 years (range, 47–82). The average duration of clinical follow-up was 106 ± 14.6 months (range, 80–135).

### Clinical outcomes

The overall Constant score in our patient population was 82.9 ± 13.1 (range, 40–97), and the subjective global shoulder function compared to a normal shoulder (SSV) was 85.0 ± 18.1 (range, 10–100). The disability of the affected shoulder according to the DASH score was rated as 10.6 ± 17.3 (range, 0–77.5). The average VAS score for pain was 0.9 ± 1.7/10 (range, 0–7).

The active global and passive glenohumeral ranges of shoulder motion are shown in Table 1.

**Table 1:** Active total and passive glenohumeral range of motion at an average 106 months follow-up

	active total range of motion in degree			passive glenohumeral range of motion in degree		
	mean ± SD	minimum	maximum	mean ± SD	minimum	maximum
Flexion	159.8 ± 13.8	110	180	92.1 ± 6.3	70	100
Abduction	155.9 ± 20.1	110	180	87.o ± 7.9	70	100
External rotation	34.8 ± 15.3	0	60	40.4 ± 13.1	15	60
Internal rotation	88.9 ± 7.9	80	110	x	x	x

*SD* Standard deviation

### Imaging

X-ray imaging data were available for all 28 shoulders (in 27 patients) who presented for follow-up. Grade I radiolucency lines (RLLs) were found in 4 shoulders (14.3%). Most lucencies appeared in Zone 5 (3 shoulders). Each shoulder had RLLs in zones 2, 4 and 7. RLLs > 1 mm were not observed.

**Fig. 2:**
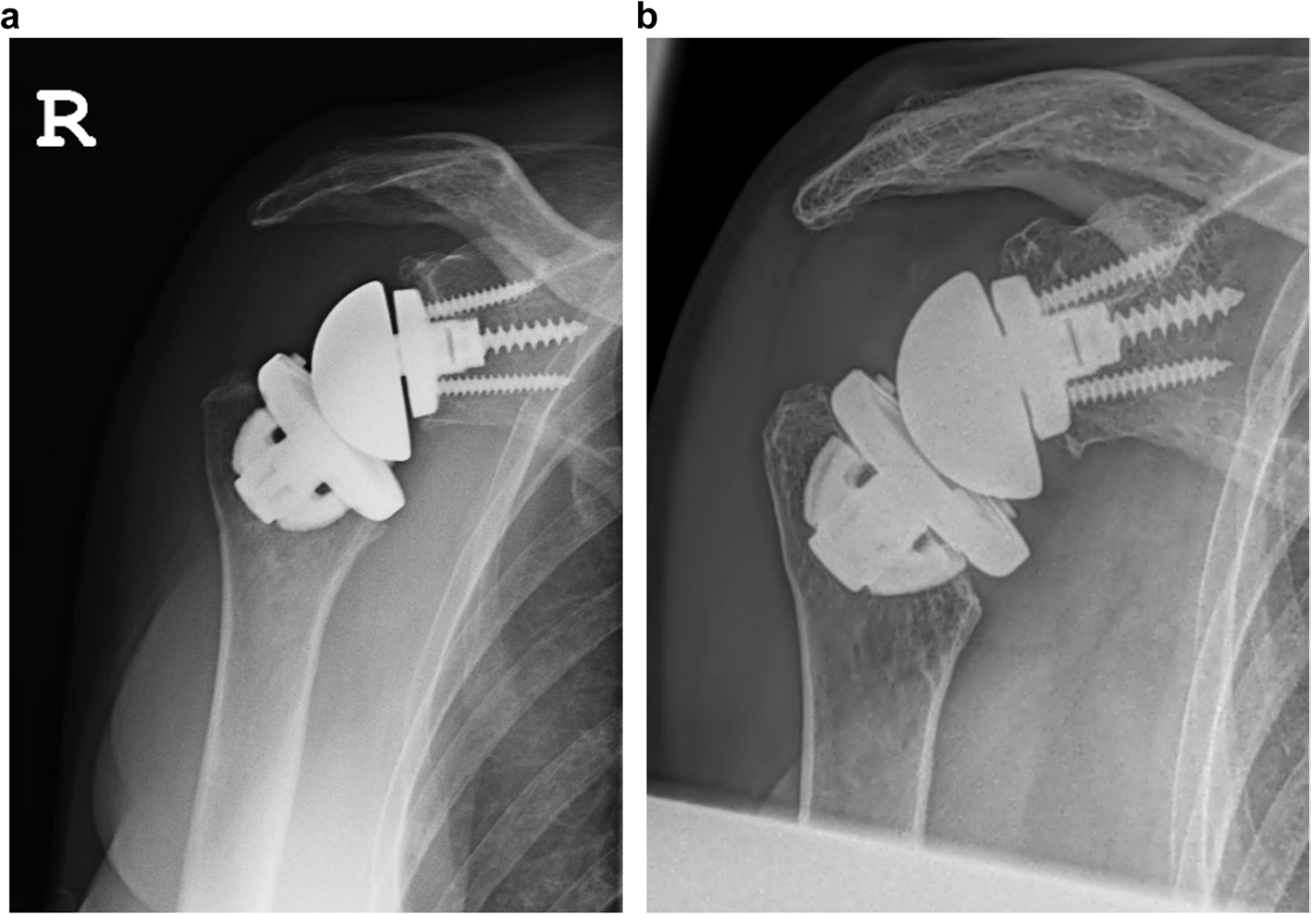
X-ray imaging postoperatively (**a**) and at the 128-month follow-up (**b**) showing secondary resorption in the calcar region

**Fig. 3:**
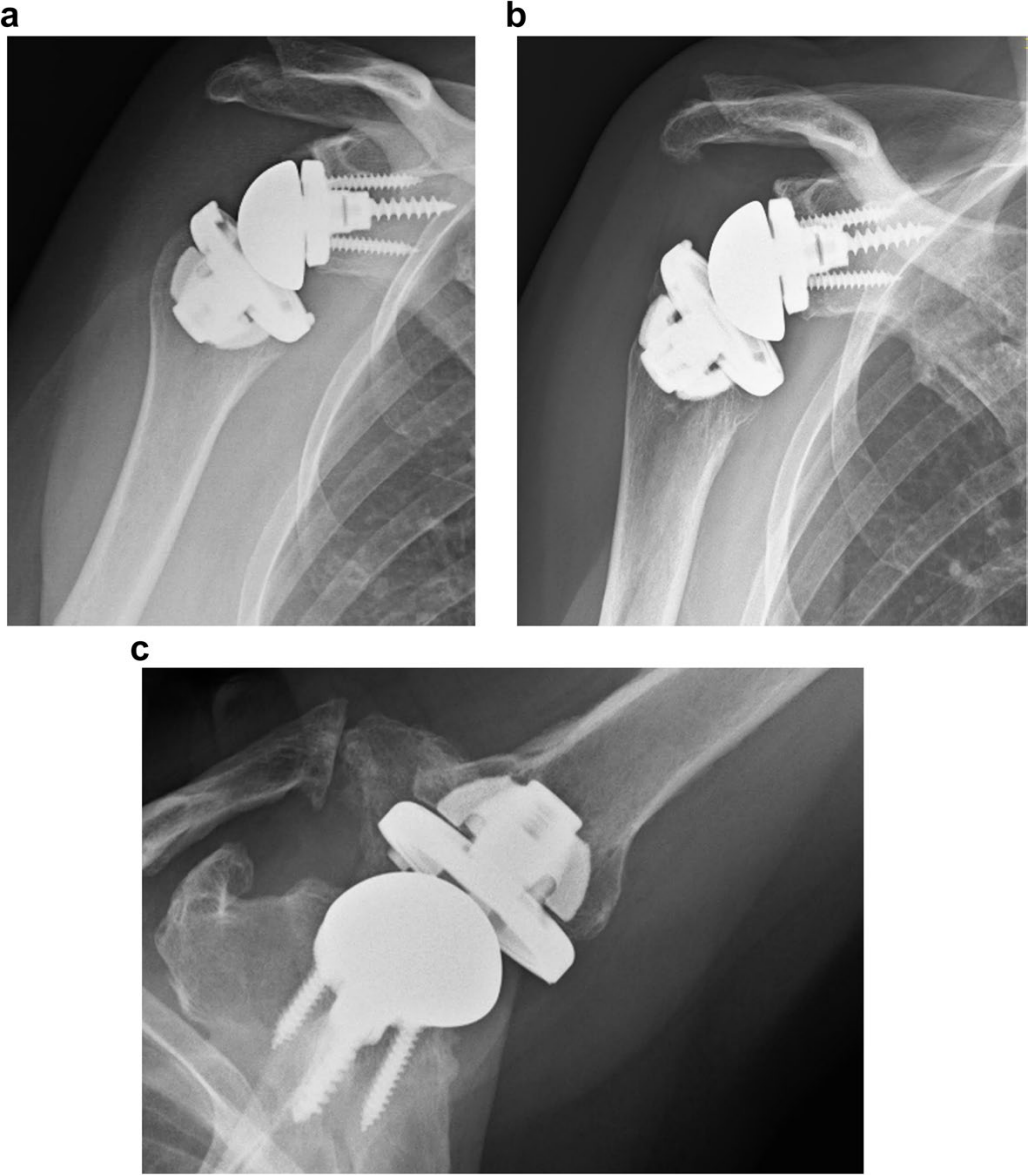
X-ray imaging postoperatively (**a**) and at follow-up (**b**/**c**) after 119 months showing significant resorption at the greater tuberosity of this 90-year-old woman at follow-up

Partial resorption was found in 3 shoulders. In two shoulders, the calcar region was affected by bony resorption. (Fig. 2a/b) The third shoulder showed resorption at the greater tuberosity at 119 months follow-up. Clinically, a positive lag sign for external rotation was found in this patient. The X-rays of this 90-year-old woman, however, did not show any loosening at follow-up. (Fig. 3a-c) Stress shielding and bone resorption at the humeral diaphysis did not occur during long-term follow-up.

### Complications and revisons

**Fig. 4:**
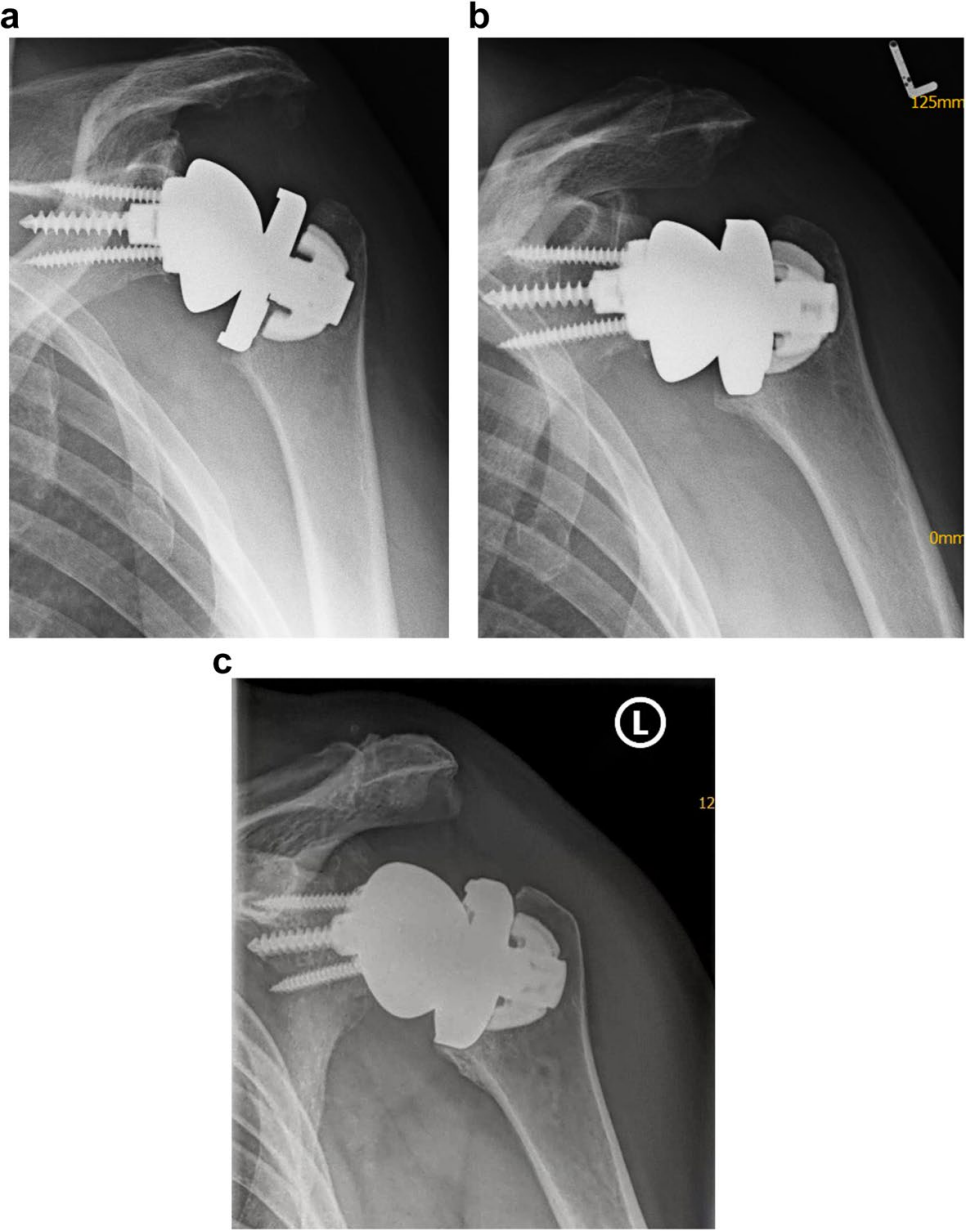
Example of a male patient (61 years old at surgery) with secondary varus displacement: postoperative imaging (**a**), humeral component displacement at 3 months post-operative imaging (**b**) and full integration at 100 months post-operative follow-up (**c**)

Two patients experienced early varus displacement of the humeral tray at 2 and 3 months after surgery. In both cases, the Nano anchor fully reintegrated with moderate displacement without the need for revision. (Fig. 4a-c)

**Fig. 5:**
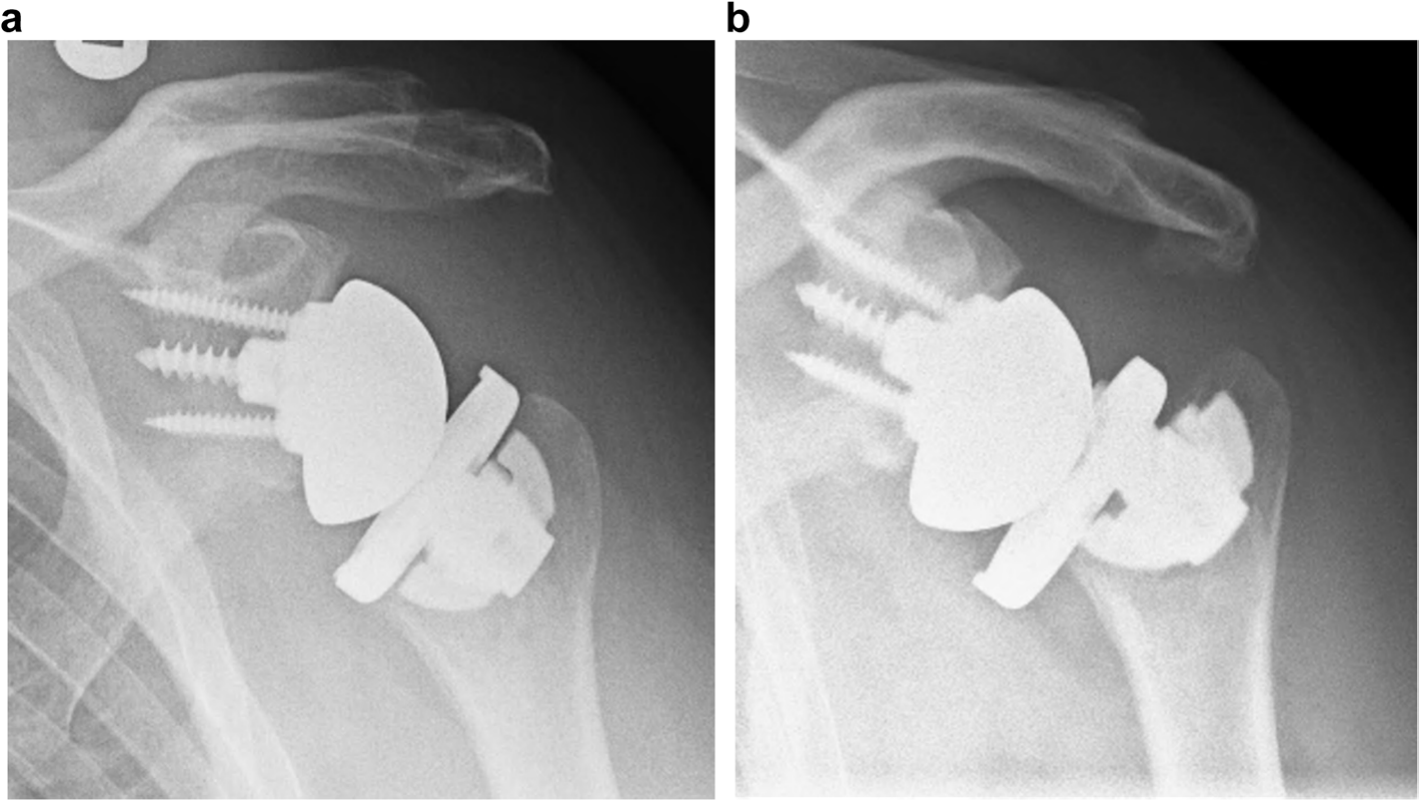
X-ray imaging of a patient with implant failure. Postoperative imaging (**a**) and breakage of the central peg of the humeral tray with disengagement from the Nano anchor 3 years after primary implantation (**b**)

**Fig. 6:**
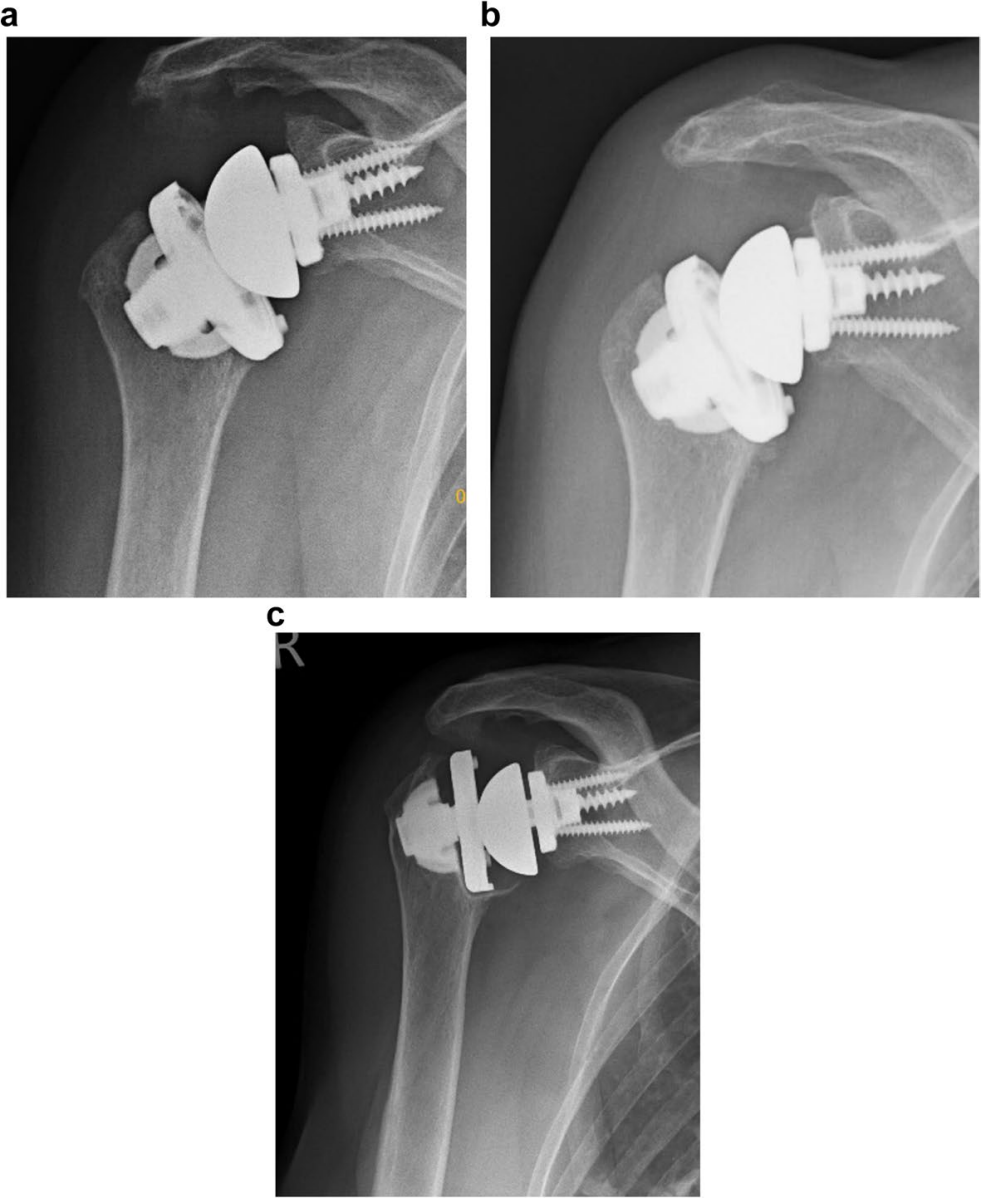
Postoperative X-ray imaging (**a**) and slight displacement of the Nano anchor 6 months after surgery (**b**). Severe atraumatic displacement, subsidence and peg breakage 7 years after surgery (**c**)

Overall, 4 shoulders (in 4 patients) were revised to a stemmed implant. In two shoulders, the peg of the humeral tray failed, and the tray disengaged from the Nano anchor. In the first shoulder, peg breakage occurred 3 years after implantation without any trauma. (Fig. 5a/b) The second patient experienced early varus displacement on postoperative imaging of the implant 6 months after surgery but did not experience any loosing during close follow-up visits and good clinical function. Seven years after surgery, however, the patient complained of a sudden loss of function without any trauma. X-rays revealed further varus displacement, severe Nano anchor subsidence and breakage of the peg of the humeral tray (Fig. 6a-c).

One patient experienced early septic loosening and subsidence of the implant. Microbiological samples obtained during one-stage revision 4 months after initial surgery revealed infection with Cutibacterium acnes. All patients were revised by the senior author to a standard size cemented stem without any further intraoperative or postoperative problems.

Another patient suffered a traumatic periprosthetic fracture 11 years after implantation following a fall and was externally revised to a stemmed implant. During the telephone interview, the patient reported satisfactory subjective shoulder function and occasional pain before the trauma. X-rays taken during postoperative follow-up revealed grade 1 notching according to Sirveaux et al. [21] and an insufficient fracture of the acromion.

During radiological follow-up, grade 1 notching was found in 5 shoulders (17.9%), and no higher levels of notching were found. One patient experienced an acromion fracture (3.6%).

Further complications included fracture of the upper part of the greater tuberosity in one shoulder on postoperative imaging with slight posterior displacement. The fragment, however, did not show further dislocation during follow-up visits and remained in stable pseudoarthrosis. No loosening of the Nano implant was found.

Furthermore, 3 patients experienced postoperative neurological deficits in the brachial plexus. Two patients presented with an isolated axillary nerve lesion. In both patients, neurological function fully recovered within the first year after surgery. The third patient experienced a lower brachial plexus stretch injury, which affected mainly the median nerve. At the 90-month follow-up, incomplete remission was observed, with persistent sensory deficits in the median nerve supply area.

Without considering grade 1 notching, complications occurred in 13 out of 35 shoulders (37.1%), and the revision rate was 11.4%.

## Discussion

In the present study, we found good clinical and radiological outcomes at long-term follow-up for the stemless Comprehensive Nano rTSA, but high rates of implant-associated complications and revision were reported.

Most studies on stemless rTSA provide only a short follow-up of 2–4 years, which may limit direct comparison to our study results. To our knowledge, Beck et al. [9] published the only long-term study to date on stemless rTSA. The authors evaluated 29 shoulders that were treated with the TESS (Total Evolutive Shoulder System, Biomet France) rTSA system at 101.6 months after surgery. The mean age of the patients was 72.4 years. However, only 12 of these 29 patients were treated with a stemless implant. Despite a reported revision rate of 17.2%, no implant-associated complications or radiolucencies at the final follow-up were reported. The clinical outcomes of the mixed cohort of 29 patients showed a Constant score of 60.5, a DASH score of 28.9, a VAS score of 1.4 and therefore inferior functional outcomes compared with those of the current study (Constant score of 82.9, DASH score of 10.6, and VAS score of 0.9).

Ballas and Beguin [22] published mid-term results of the stemless TESS rTSA in 59 patients at 58-month follow-up. The clinical outcome revealed an active flexion of 140 degrees and an overall Constant score of 62 points; therefore, the outcomes were again inferior to those of the current study. One patient underwent revision at the humeral side to a stemmed implant due to displacement of the humeral corolla 3 days after initial surgery. At the latest follow-up, no humeral radiolucency, migration, or loosening of the reverse humeral cup was observed.

Comparative studies on stemless and stemmed rTSA have shown satisfactory outcomes for both implants and overall comparable complication and revision rates at short-term follow-up [23, 24]. A’Court et al. [24] retrospectively evaluated 33 stemless Lima SMR rTS (San Daniele del Friuli, Italy) and compared the outcomes to those of 33 stemmed Lima SMA rTSA. The clinical outcomes rated by the Oxford Shoulder score and the American Shoulder and Elbow Surgeons score were comparable in both groups, while 3 patients in the stemless group had to undergo revision within the 2-year follow-up for chronic infection, periprosthetic fracture and gross implant instability, respectively. Interestingly, the authors also reported 2 patients in the stemless group who experienced radiologic subsidence of the humeral implant with full reintegration under conservative treatment and no need for revision.

Moroder et al. [20] evaluated a group of 24 stemless TESS rTSA and compared them to a matched control group following conventional stemmed DELTA Xtend rTSA (DePuy Synthes, Warsaw, IN, USA). At short- to midterm follow-up, the authors reported comparable clinical outcome scores, ranges of motion, complications and revisions.

In a current systematic review on stemless rTSA, Hatta et al. [25] published the pooled outcomes of 637 shoulders from 14 studies. Most studies in this meta-analysis had a short-term follow-up of less than 4 years [4, 15, 23, 24, 26–29]^,^ and some study outcomes were limited to younger patients with an average age at surgery of 65 years or less [24, 26]. The average follow-up, however, was 40.4 months. The pooled Constant score outcome averaged 62.8 points, ranging from 56.9 to 83.1 points. The clinical results of our patient population, as judged by the Constant score, are within the upper range and clearly superior to the average reported outcomes. The pooled incidence of overall complications in this meta-analysis was 14.3%, with substantial heterogeneity across studies ranging from 0 to 34.6%. As so often in clinical research, this seems to be related to the individual interpretation and definition of a complication by the individual authors. The revision rate, on the other hand, was more homogenous, averaging 6.3% of all patients. The most common indication for revision was instability (19.5%), followed by periprosthetic humeral fracture (13.4%) and humeral malposition/displacement/migration (13.4%).

In the present study, no patients experienced instability or dislocation of the implant, and fractures were less common than previously reported. However, we observed migration/subsidence in 4 of our patients. While in one patient, this was clearly associated with an infection, 3 patients experienced migration of the humeral implant due to mechanical loads. In two patients, the implant reintegrated fully without any loosening or radiolucency on follow-up X-rays. Another patient showed early migration at 6 months after surgery. During close further follow-up, the Nano anchor appeared to have fully reintegrated, and the patient showed good clinical function. Seven years after surgery, however, the patient suffered a sudden loss of shoulder function. Further subsidence of the Nano anchor and failure of the peg of the humeral tray could be recognized on X-rays. Overall implant failure of the peg of the humeral tray was observed twice. In the second patient, atraumatic failure occurred 3 years after surgery. The company reacted to these circumstances and changed the material of the tray from titanium to cobalt-chrome.

Overall, the incidence of implant-associated complications is high within our patient population. One explanation may be the early application of this stemless “onlay” implant. As the first user of this system worldwide, there was no clinically based evidence for this type of implant.

Furthermore, the high complication rate may be associated with the “onlay” design of the Comprehensive Nano rTSA. The “Onlay” system has a longer lever arm with a potential risk of seesaw motions at the implant–bone interface [7]. In “inlay” designs, on the other hand, the implant is completely embedded into the metaphyseal bone below the humeral head cut, and the forces are transferred directly to the bone.

Most stemless rTSA implants on the market follow an “inlay” design. Beside the Comprehensive Nano rTSA, only the EasyTech (FX Solutions, Viriat, France) stemless rTSA system follows an “onlay” concept. In 2023, the only available study on this implant was published [4]. In this large multicenter 24-month follow-up study of 115 shoulders, the authors noted an average Constant score of 61.8 points and an SSV of 77.5, again showing inferior outcomes compared with the patients in our study group. However, complications and reoperations were lower at this short-term follow-up, with an overall complication rate of 17.2% and an implant revision rate of 7%. Yet the authors of the study concluded that surgeons should proceed with caution when using this stemless “onlay” implant. Again, comparing our long-term outcomes with 24-month short-term outcomes is not possible because many complications in our population occurred after 24 months.

Notably, the number of iatrogenic neurological lesions in our population was greater than that reported in the literature (8.6%). In the previously cited review article of Hatta et al. [25] on stemless rTSA, an incidence of 0.5% was reported. While this number, on the other hand, appears to be low, a current publication from a specialized shoulder unit reported a neurological complication rate of 2.6% in their patient population of 1309 rTSAs [30]. Again complications may be undetected or underreported by some authors, but the reason for the high incidence in our study remains unclear. Compared with an “inlay” design, a “onlay” implant may create more distalization and therefore more tension on the plexus, which has been reported as a contributing factor for neurologic injuries [31]. To our knowledge, however, no clinical study has reported a higher incidence of “onlay” designs. A further explanation is the application of a hydraulic arm positioner (Spider Limb Positioner, Smith & Nephew, London, England), which the senior author started to use in 2013. Excessive traction during the early application of the limb positioner for shoulder arthroplasty may also explain the high rate of traction injuries in our series [32]. 

There are several limitations of the present study that need to be considered. First, owing to the long follow-up of the current study, 34% were lost to follow-up. The long-term follow-up of an elderly population inevitably results in a high drop-out rate due to immobility, comorbidities or death, increasing the possibility of selection bias. Second, we retrospectively reported the outcomes of a small patient population without a control group. Third, this is a preselected group of patients who were selected by the surgeon as candidates for a stemless shoulder prosthesis and did not correspond to the average patient population for the rTSA. The results should be interpreted accordingly. Fourth, comparisons with published data on other stemless rTSA systems are limited because of a lack of long-term studies in the literature. Although the comprehensive Nano rTSA system was withdrawn from the market in 2017 and may no longer be used in a reverse configuration, we believe that the knowledge gained from the current study may be transferable to other systems and will help to further improve stemless rTSA in the future.

In the present study, however, we present long-term follow-up data on stemless rTSA, which is currently a hot topic in shoulder arthroplasty and will probably remain so in the near future.

## Conclusion

Compared with published data on stemmed and stemless rTSA, the comprehensive Nano rTSA system in the current study has comparable or even superior clinical outcomes at long-term follow-up. The rates of implant-associated complications and revision, however, are high compared with those reported in the literature.

## Data Availability

The datasets used and/or analysed during the current study are available from the corresponding author on reasonable request.

## Abbreviations

rTSA: Reverse total shoulder arthroplasty
aTSA: Anatomic total shoulder arthroplasty
DASH score: Disabilities of the Arm, Shoulder and Hand score
SSV: Subjective shoulder value
VAS: Visual analog scale
RLL: Radiolucency line
TESS: Total Evolutive Shoulder System
